# Nonspecific Effects of the Bacillus Calmette-Guérin Vaccine in Portuguese Children Under 5 Years of Age: Protocol for a Population-Based Historical Birth Cohort Study

**DOI:** 10.2196/55332

**Published:** 2024-03-14

**Authors:** Ines Fronteira, Matilde Pacheco, Frederik Schaltz-Buchholzer, Paulo Ferrinho

**Affiliations:** 1 NOVA National School of Public Health Public Health Research Center, Comprehensive Health Research Center NOVA University Lisbon Portugal; 2 Global Health and Tropical Medicine, GHTM, Associate Laboratory in Translation and Innovation Towards Global Health, LA-REAL Instituto de Higiene e Medicina Tropical, IHMT Universidade Nova de Lisboa, UNL Lisbon Portugal; 3 Instituto de Higiene e Medicina Tropical Universidade Nova de Lisboa Lisbon Portugal; 4 Bandim Health Project OPEN, Department of Clinical Research University of Southern Denmark and Odense University Hospital Odensen Denmark

**Keywords:** BCG, Bacillus Calmette-Guérin vaccine, policy, Portugal, nonspecific effects, vaccines, heterologous immunity

## Abstract

**Background:**

The Bacillus Calmette-Guérin vaccine (BCG) against tuberculosis (TB) shows beneficial nonspecific effects, which are likely related to innate immune training. Until 2016, a single BCG dose was administered to all newborns in Portugal. In July 2016, a clinical guideline established that only children under 6 years belonging to high-risk groups should receive BCG. This might have prevented nonvaccinated children from developing trained immunological responses as effectively as BCG-vaccinated children.

**Objective:**

This study aims to investigate if there is variation in TB-related and all-cause mortality, and severe, moderate, or mild morbidity in children under 5 years of age, and whether such variation might be explained by the BCG vaccination policy change in 2016.

**Methods:**

This population-based historical birth cohort study includes children under 5 years of age born in Portugal between July 1, 2010, and June 30, 2021. Newborns with low birth weight, premature status, or known or suspected HIV infection are excluded. The follow-up period is until the completion of 5 years of age or the end of follow-up (June 30, 2021). The study will use secondary data from the National Health Service user registry, death certificate database, vaccination registry, communicable diseases surveillance system, TB surveillance system, diagnosis-related group information system for hospital admissions and emergency department visits, and primary health care information system. The data will be linked. Primary outcomes include person-time incidence rates of death (all causes and TB), TB diagnosis, and all causes and some specific causes of severe, moderate, or mild morbidity, and the incidence rate ratio of nonvaccinated to BCG-vaccinated children. We will compare the probability of surviving the first and fifth years of life or of not having severe, moderate, or mild morbidity during the follow-up period according to exposure (BCG vaccinated or nonvaccinated, number of doses, and time from birth until the first dose), using the log-rank test for assessing differences in survival rates between exposed and nonexposed children and hazard ratios for quantifying the differences. Moreover, we will perform a proportional hazards regression analysis.

**Results:**

Ethics approval has been obtained. In March 2022, database owners were contacted to present the project and discuss the request for data. A unique identifier will be used. In July 2023, a process of redefinition of the variables per database was initiated. Data were received in October and November 2023. In November 2023, further work was conducted. By April 2024, we expect to start analyzing the full data set.

**Conclusions:**

The results will contribute to the accumulating body of knowledge and might have relevance to guide global BCG vaccination policy. Data linkage can contribute to a swifter mechanism to use available health data to conduct population-based studies and inform policy decision-making.

**Trial Registration:**

ClinicalTrials.gov NCT05471167; https://clinicaltrials.gov/study/NCT05471167

**International Registered Report Identifier (IRRID):**

DERR1-10.2196/55332

## Introduction

### Background

Immunization has led to a significant decrease in mortality in children under 5 years of age. Several studies have demonstrated that the reduction in mortality and morbidity due to vaccination extends beyond the targeted infections. This seems to result from broader nonspecific effects (also termed heterologous immunity) stemming from the synergistic effects of several live vaccines [1-4]. The vaccines demonstrated to have nonspecific effects include the Bacillus Calmette-Guérin vaccine (BCG), polio vaccine, and measles vaccine [5]. Overall, the nonspecific effects of vaccines vary according to age, sex, time to administration, last vaccine administration, previous and concurrent infections and immunizations, interval between vaccines, genetic factors, nutritional state, season, and co-administration of other immunomodulating agents [1].

BCG is a live attenuated vaccine against tuberculosis (TB), which is administered to newborns ideally at birth or before the seventh day of life. Despite its low efficacy for preventing primary infection or reactivation of latent pulmonary infection and TB infection, BCG is effective against childhood tuberculous meningitis and miliary disease [6]. Hence, BCG at birth is recommended in settings with a high incidence of TB, and it is parsimoniously administered in countries with a low incidence and has been removed from routine vaccination schemes in high-income countries with a low TB incidence [7].

BCG was introduced in the 1920s in Sweden, where it quickly became apparent that the all-cause mortality was lower in BCG-vaccinated children than in those not vaccinated [8]. In England and the United States, the same pattern was observed between the late 1940s and early 1960s, where a reduction in mortality from diseases other than TB was estimated in 25% of vaccinated children (95% CI 6%-41%) [1,9]. Later studies showed the same pattern in infant mortality and morbidity [10], as well as in morbidity in the neonatal period [11]. Furthermore, BCG is used as a standard treatment for carcinoma of the bladder and influences the natural history of infectious and neoplastic diseases [1]. More recently, the effect of BCG was tested in viremia after SARS-CoV-2 exposure [12].

Further studies in children from Denmark and Greenland failed to show a decrease in hospitalization due to infectious diseases in vaccinated children [13,14]. On the contrary, a cohort study of children from 19 different countries revealed that BCG-vaccinated children under 5 years of age had a lower risk of suspected acute lower respiratory infection [15]. In Spain, hospitalization rates due to respiratory infection were lower in BCG-vaccinated children, with an attributable fraction of 32% for children under 1 year of age, and a similar finding was noted for sepsis, with an attributable fraction of 53% [16].

A systematic review concluded that BCG has a beneficial effect on mortality in children, with differential effects according to age (earlier administration of the vaccine is associated with greater effects) [5]. Nevertheless, there has been a call for more research on the nonspecific effects of BCG [5,17]. Currently, studies are seeking to further investigate the nonspecific effects of BCG [18] in terms of the effects on allergy and infection [7], severe morbidity, nonaccidental hospital admission, and all-cause consultation in children under 5 years of age [19].

### BCG Vaccination in Portugal

In Portugal, BCG began to be routinely administered to all newborn children in 1965. Until 2016, a single BCG dose was administered to all newborns, typically before discharge from the maternity ward (most births in Portugal are hospital-based) or as early as possible thereafter [20].

In June 2016, a clinical guideline established that only children under 6 years of age who belong to high-risk groups (those originating from countries with high TB incidence; having contact with active cases or persons under prophylaxis; having HIV-positive mothers; having parents with a alcohol or drug abuse problem and antecedents of TB; having parents who have been in prison in the last 5 years; living in high-risk TB communities; or traveling to high-incidence countries) should be vaccinated as close to birth as possible [21]. Some of these risks were not considered individually but by proxy, with some parishes (freguesias) thus considered high risk for the criteria defined.

In 2016, 42% of children under 1 year of age had received BCG (compared to 98% in the previous year) [20]. Between 2016 and 2018, the incidence of TB increased by 16% in children under 1 year of age (from 7.0 to 8.1 per 100,000 inhabitants) and 192% in children aged 1 to 5 years (from 2.6 to 7.6 per 100,000 inhabitants) [20].

Between the adoption of the high-risk strategy for BCG vaccination in 2016 and 2018, the infant mortality rate (IMR) remained stable at 3.2‰ to 3.3‰, with important variations between the considered years [20]. In 2019, there was a decrease to 2.8‰. The mortality rate in children under 5 years of age decreased from 3.4‰ in 2016 to 3.0‰ in 2019, but not constantly in this period (eg, during 2018 it was 3.5‰) [20]. No death in children under 5 years of age has been attributable to TB since the implementation of the high-risk strategy for BCG [20]. Nevertheless, the number of severe cases of TB in children under 5 years of age decreased in 2020 compared to 2018 (4 cases) and 2019 (7 cases), with just 1 case registered in a child without BCG vaccination [22].

### Evaluation of the BCG Vaccination Policy

Since the implementation of the high-risk strategy for BCG in 2016, no assessment has been conducted regarding the impact of the BCG policy shift on severe, moderate, or mild morbidity and on mortality in children. It is a common problem that vaccination practices are changed without evaluating the overall health effects of the change and that the nonspecific effects of vaccines are not included in the considerations regarding vaccine policies.

In 2021, the first cohort of children not vaccinated for BCG completed the fifth year of life, and evidence is needed regarding the impact of this strategy, especially given fluctuations in the IMR, a decline in the TB notification rate not accompanied by a decline in the incidence, a higher TB incidence in greater metropolitan areas, and a high median number of days from symptoms to diagnosis [22].

No data have been gathered on overall hospital admissions and morbidity patterns. Similarly, no data have been compiled on the BCG strains used in the country and their differences in terms of efficacy. This evidence will have a bearing on the continuity of the current strategy.

The need for more and better evidence is paramount, especially given that besides deciding on the inclusion of BCG in the country’s immunization plan, arguments on the nonspecific effects of vaccines (and not only of BCG) can help overcome vaccine hesitancy. In high-income countries, including Portugal, vaccine hesitancy has been responsible for a decline in coverage rates and for the re-emergence of severe cases of vaccine-preventable diseases such as measles [23,24].

### Hypothesis

Given the described effects of BCG on overall mortality, immune and atopic conditions including asthma, and incidence of respiratory tract infections; its nonspecific protection against nonrelated pathogens; and its protective effects that are apparent shortly after immunization and sustained for at least 1 year [2,25], we hypothesize that some of the variations in mortality among children under 5 years of age, in the incidence of TB, and in severe, moderate, and mild morbidity among children under 5 years of age might be partially explained by a reduction in the coverage rate of BCG since 2016. The reduction in the coverage rate of BCG, which resulted from only high-risk children receiving BCG at birth, might have prevented nonvaccinated children from developing trained immunological responses as effectively as BCG-vaccinated children.

### Objectives

We aim to investigate the incidence of the specific and nonspecific effects of BCG by comparing the incidences of TB disease and infection; mild, moderate, and severe morbidity; and mortality in the first 5 years of life among children born in Portugal between 2010 and 2021, according to their BCG status.

## Methods

### Study Design

This population-based historical (retrospective) birth cohort study will compare the incidence of all-cause mortality (including due to TB disease); TB disease; and severe, moderate, and mild morbidity between BCG-vaccinated and nonvaccinated children born between July 1, 2010, and June 30, 2021.

### Setting

This study will investigate the specific and nonspecific effects of BCG based on the shift in the BCG vaccination policy that occurred in Portugal in July 2016, when a consistently low incidence of TB led to the revision of the National Immunization Plan. Since then, BCG has been administered only to newborns considered to be at higher risk of TB infection, thus creating an opportunity to compare BCG-vaccinated and nonvaccinated children.

The approach adopted by Portuguese public health authorities to define the high risk for TB was based on geographical risk, with, for instance, all children born in some parishes in the metropolitan areas of Lisbon and Porto being offered BCG independent of socioeconomic status, country of origin, or vulnerability. We believe that this type of risk definition has guaranteed enough variability between BCG-vaccinated and nonvaccinated children, thus allowing comparisons of health outcomes between exposed and nonexposed children.

### Participants

**Figure 1:**
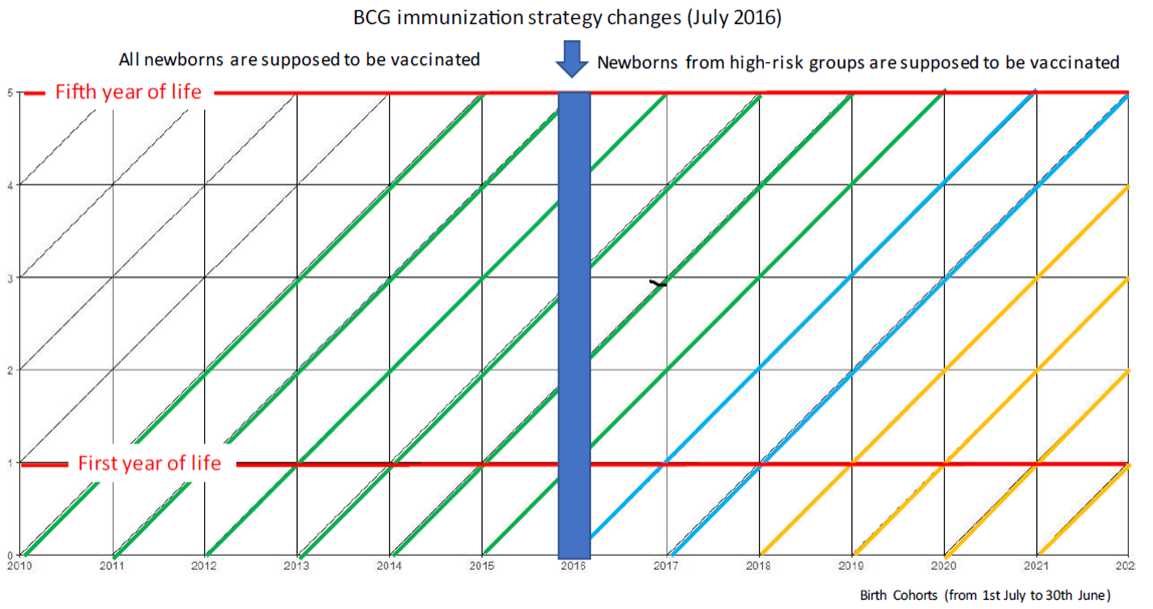
Lexis graph. BCG: Bacillus Calmette-Guérin vaccine.

**Figure 2:**
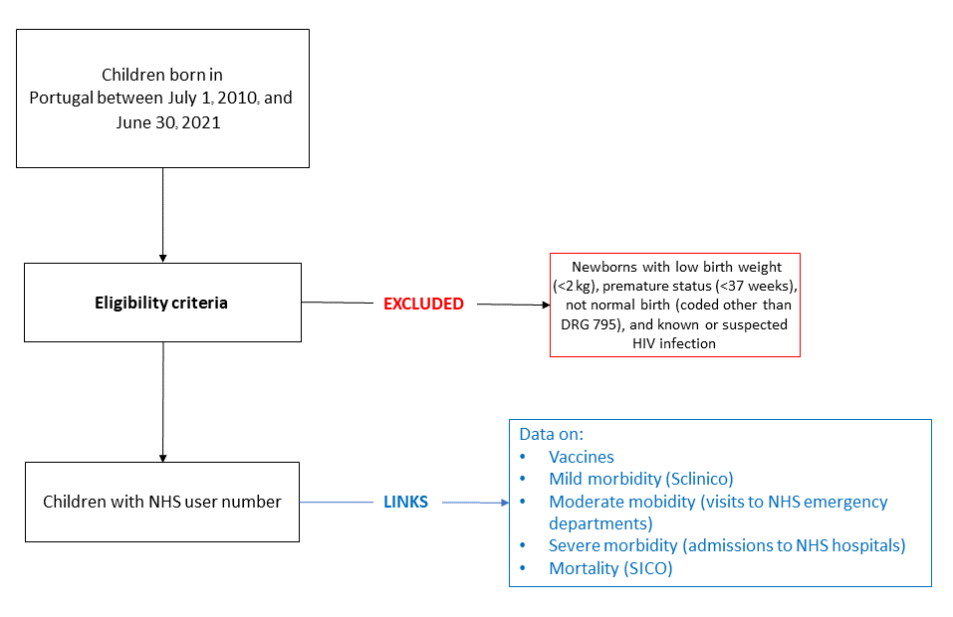
Flowchart. DRG: diagnosis-related group; NHS: National Health Service.

The study will include children under 5 years of age born and registered in Portugal between July 1, 2010, and June 30, 2021. We have chosen this period because it will allow us to (1) compare the cohorts before and after the change in the BCG immunization strategy (Figure 1); (2) characterize children not vaccinated with BCG and their health outcomes (when universal BCG at birth was recommended); and (3) compare children exposed and not exposed to BCG after the 2016 change in the BCG vaccination strategy.

#### Eligibility Criteria

The study will include children born alive in Portugal (births coded as diagnosis-related group [DRG] 795 [normal newborn]).

#### Exclusion Criteria

The study will exclude newborns who have a weight of <2 kg, are premature (<37 weeks of gestation), or are known or suspected to have HIV infection.

The follow-up period will be until the completion of 5 years of age or the end of follow-up (June 30, 2021) (Figure 2).

### Data Sources

The study will be based solely on secondary data. The data sources are the National Health Service (NHS) user registry (RNU), Death Certificate Information System (SICO), vaccination registry, communicable diseases surveillance system (Sistema Nacional de Vigilancia Epidemiologica [SINAVE]), surveillance information system for TB (Svig-TB), DRG information system for hospital admissions and visits to the emergency department (Base de Dados de Morbilidade Hospitalar [BDMH]), and primary health care (PHC) information system (SClínico) of children born between July 1, 2010, and June 30, 2021.

The birth registry database (RNU) contains data on gender, age, PHC center where the child is to be followed up, nationality, and place of residence. The SICO database provides data on the cause and date of death. The vaccination registry includes data on the vaccines administered, date of vaccination, and place of residence. The BDMH database gathers data on hospital admissions and emergency department visits to NHS hospitals, including the date of admission and discharge, diagnosis, type of admission, motive for admission, weight at birth, DRG code, major diagnostic category (MDC) code, complications of pregnancy and delivery, and type and place of delivery. Data from admissions at private hospitals are not included. The SClínico database contains data on socioeconomic status and visits (including motive and diagnosis) to NHS PHC units. Svig-TB is a database for notification and follow-up of TB cases, and SINAVE is the general surveillance information system for communicable diseases, including TB, and regarding TB, it contains data on symptoms at presentation, date of diagnosis, type of treatment, other diseases before TB, risk groups, and outcomes of the infection.

### Measurements

Exposure is defined as having received the BCG vaccine during the first year of life and is measured using the vaccination registry. Variables include BCG (yes or no, and number of doses) and time from birth to BCG (in days).

### Outcomes

#### Primary Outcomes

In this study, the primary outcomes for BCG-vaccinated and nonvaccinated infants and children under 5 years of age include the incidence of all-cause death and death due to TB; the incidence of mild, moderate, and severe morbidity; and respective relative risks.

The primary outcomes will be measured as person-time incidence rates of death (all causes and TB); TB diagnosis; and severe, moderate, and mild morbidity in the follow-up period, and the incidence rate ratio of nonvaccinated to BCG-vaccinated children (Table 1). For the outcome of death, data will include death (yes or no), age at the time of death (in days), and cause of death (eg, diseases of the respiratory system and diseases of the skin and subcutaneous tissue). Data on TB diagnosis will include confirmed case (yes or no), presentation of TB, age at diagnosis (in days), duration of treatment, and outcomes of treatment. Severe and moderate morbidity will be measured based on hospitalization and visits to the emergency department of the NHS. Outcomes will be characterized by age at presentation (in days), frequency of admissions or visits to the emergency department, length of stay for hospital admission (in nights), and MDC code for hospitalization and emergency department visits. We will include the outcome of hospital admission (discharged alive or died) in case fatality risk analyses. The outcome of mild morbidity is defined as contact with a medical doctor or nurse at a PHC unit owing to disease or ill health during the follow-up, as well as contact related to a recommended child surveillance scheme or other reasons besides health. It will be characterized through visits to the PHC center owing to disease or ill health (yes or no, and number), age at the visits (in days), and diagnosis (International Classification of Primary Care-2 [ICPC-2]). Mortality and morbidity pertaining to external causes and accidents will not be included in the analysis.

**Table 1:** Predictable analysis per cohort and outcome.

Analysis^a^	Outcomes
Exposed children in birth cohorts 2010 to 2015 compared to nonexposed children in birth cohorts 2010 to 2015, 2016, 2017, 2018, 2019, 2020, and 2021	Primary outcomes in the first year of life
Exposed children in birth cohorts 2010 to 2015 compared to nonexposed children in birth cohorts 2010 to 2015, 2016, and 2017	Primary outcomes in the first 5 years of life
Exposed children in birth cohorts 2016, 2017, 2018, 2019, 2020, and 2021 compared to nonexposed children in birth cohorts 2016, 2017, 2018, 2019, 2020, and 2021	Primary outcomes in the first year of life
Exposed children in birth cohorts 2016 and 2021 compared to nonexposed children in birth cohorts 2016 and 2017	Primary outcomes in the first 5 years of life
Nonexposed children in birth cohorts 2010 to 2015 compared to nonexposed children in birth cohorts 2016 to 2021	Secondary outcome profile of nonvaccinated children before and after the 2016 BCG^b^ vaccination change
Children in birth cohorts 2010 to 2015, 2016, and 2017	Secondary outcomes of the mortality and morbidity profile of children under 5 years of age, and NHS^c^ hospital and primary health care utilization profiles
Exposed children in birth cohorts 2010 to 2015 compared to exposed children in birth cohorts 2016, 2017, 2018, 2019, 2020, and 2021	Secondary outcomes of the mortality and morbidity incidence according to BCG strains administered per cohort in the first 5 years of life and first year of life
Exposed children in birth cohorts 2010 to 2015 compared to exposed children in birth cohorts 2016 and 2017	Secondary outcomes of the mortality and morbidity incidence according to BCG strains administered per cohort in the first 5 years of life

^a^Between 2010 and 2015, Bacillus Calmette-Guérin vaccine (BCG) coverage varied between 98.2% and 98.4%. In 2016, BCG coverage was 41.6%.

^b^BCG: Bacillus Calmette-Guérin vaccine.

^c^NHS: National Health Service.

#### Secondary Outcomes

The secondary outcomes include the mortality and morbidity profiles of children under 5 years of age between 2010 and 2021, including causes of death and morbidity, the profiles of nonvaccinated children, and the NHS hospital and PHC utilization profiles of children under 5 years of age before and after 2016 (Table 1).

### Statistical Methods

Through data linkage, we will create a single database that links data from the birth registry, SICO, vaccination registry, SINAVE, Svig-TB, BDMH, and PHC database (SClínico) to reconstruct chronological sequences of morbidity and mortality events from birth until the completion of 5 years of life or the end of follow-up (for the 2018 cohort onwards).

In each of the databases, we will identify the data needed and whether a unique identifier (UI) exists and can be provided with the data set. After having access to the requested data and in case a UI is provided (and common to all databases), we will combine information based on that UI.

In case a UI is not provided, we will link data from several data sets using a range of proxy identifiers (eg, date and place of birth, sex, and place of residence) to identify probable matches.

The type of data linkage method to be used will depend on the type and quality of the linkage variables available in the data sets. However, we anticipate having to use a combination of deterministic and probabilistic methods. Before applying linkage methods, we will clean and standardize the data, thus identifying and removing errors and inconsistencies. Given the expected size of the data sets, we will then select sets of block attributes (eg, sex, date of birth, and initials) and compare record pairs with the same matched attributes within blocks. Subsequently, record pairs will be compared for each linkage variable, and an agreement score will be computed. This score will be used to weigh the probability of a record pair belonging to the same child [26,27].

After obtaining the final database, we will compute person-time incidence rates for primary outcomes in BCG-vaccinated and nonvaccinated children. Using the Kaplan-Meier method, we will compute and compare the probability of surviving the first and fifth years of life or of not having a hospitalization, emergency department visit, or mild morbidity episode during the follow-up period according to exposure. Additionally, we will use a log-rank test for assessing differences in survival rates between exposed and nonexposed children and hazard ratios (and corresponding CIs) for quantifying the differences. To explore the effects of several variables on the survival outcomes, we will use proportional hazards regression analysis. If missing data are below 5%, we will use complete case analysis. Otherwise, the frequency and patterns of missing data will be analyzed, and if appropriate, we will use multiple imputation techniques.

Confounders to be measured and controlled for are sex, health unit, completeness and timeliness of vaccination status and scheme, socioeconomic status, and BCG strain (over the years, administered BCG has been provided by different producers, with different strains).

### Ethical Considerations

This study has received ethical approval from the Ethics Committee of Instituto de Higiene e Medicina Tropical (IHMT) – Instituto de Tecnologia Química e Biológica António Xavier (ITQB) NOVA – NOVA School of Law (NSL) – Instituto Gulbenkian de Ciência (IGC) (11.23) [28].

The project protocol was submitted to the Ethics Committee of IHMT – ITQB NOVA (11.23), which issued a conditional authorization in July 2022 [29] that became definitive in December 2023.

## Results

This project was approved as an exploratory project by the Portuguese Foundation for Science and Technology in the 2021 competitive call for R&D projects (reference: EXPL/SAU-EPI/0067/2021). It was granted approximately €50,000 (US$ 53,869) and was started in January 2022.

During the first month of the project’s implementation, administrative and financial activities were carried out, with a summary of the project sent to members of the Scientific Advisory Board, including the planned activities.

In March 2022, database owners were contacted to present the project and discuss how to officially request the data. A document detailing all the data needed to carry out the study was prepared and sent to the Directorate General of Health. After approval from this body, Serviços Partilhados do Ministério da Saúde (SPMS) and Administração Central do Sistema de Saúde (ACSS) also had to provide authorization to access the data, with the last authorization given in October 2023.

We were informed that a UI would be provided for each database, based on the NHS user number, from the RNU, thus allowing direct linkage of records. However, some of the requested variables had to be reviewed and replaced given the Data Protection Law. In July 2023, a process of redefinition of the variables per database was initiated, with some of the variables requested not being supplied owing to legal restrictions (eg, date of birth had to be replaced by days from birth until the event and information if the child had been born before or after 2016) (Multimedia Appendix 1).

Data were received during October and November 2023. By mid-December, data from SINAVE had not yet been provided. No data were obtained from Svig-TB as we were not granted access. Some of the variables requested from this database were replaced by equivalent or proxy variables from SINAVE (Multimedia Appendix 1).

In November 2023, work was conducted in the databases already provided to restructure the databases, perform quality checks and cleaning, compute exposure variables, transform string variables into numeric variables, and perform coding.

By April 2024, we expect to start analyzing the full data set, which is expected to include around 970,000 children.

## Discussion

### Principal Findings

If the change in the BCG strategy in 2016 is proven to influence the health outcomes of children in their first year of life and during the first 5 years of life, it would further contribute to evidence that BCG primes the immune system against unrelated pathogens and even other health conditions, strengthening the arguments regarding the nonspecific effects of BCG. Additionally, it would provide evidence of the impact of the high-risk strategy for BCG vaccination and inform future decisions regarding BCG vaccination for children in Portugal and other high-income countries. The results will contribute to the accumulating body of knowledge and might have relevance to guide global BCG vaccination policy.

The use of data linkage can also contribute to a swifter mechanism to use available health data to conduct population-based studies and inform policies. The project will demonstrate the feasibility of conducting large-scale epidemiological register-based vaccine studies in Portugal.

### Limitations

The main limitation of this project involves the use of secondary data collected for purposes other than research. The RNU database, which provides the basis for creating a UI, only includes children who have a user number for the NHS. The user number is assigned to each person to identify them when accessing the services of the public health care units of the NHS. People with Portuguese citizenship obtain their user number automatically when they apply for a Citizen’s Card. Foreigners with a residence or stay permit in Portugal have to apply for an NHS user number. Most but not all infants are registered shortly after birth.

In August 2016, the Portuguese government created the program “Nascer Utente” (translated to “Born as a User”) that allowed for immediate registration in the RNU database, assigning the respective user number. This process is conducted by the hospital (public and private) where the child is born, immediately after birth [30]. We therefore expect a bias toward children born after this date in the RNU database.

The electronic registry of vaccines was started in 2017, and all data before this point had to be back introduced in the database, which could have increased the number of typing errors and other issues. We can again have a bias toward the most recent years in terms of the quality of data.

The number of deaths of children under 5 years of age might be underestimated since very young children tend not to have an NHS user number, especially before August 2016. In this case, we can also expect to have a bias in the age of children who died between July 1, 2010, and June 30, 2021, toward older ones.

The BDMH database only pertains to public hospitals. It does not include data from emergency department visits or hospital admissions in the private sector. The same is true for SClínico, which contains data from PHC centers in the NHS. Children using the private sector are not captured by this database. Additionally, the case mix (severity) of admissions in public hospitals is usually greater than that in private hospitals, which might introduce a bias toward higher case fatality rates in hospital admissions.

In 2020, 32% of Portuguese people had voluntary health insurance (in 2010, the proportion was less than 20%), and the proportion is dependent on household income, with those in higher income groups showing a higher probability of having voluntary health insurance. As such, we expect a bias toward lower income groups, especially in the last years of the study period [31].

### Comparison With Prior Work

This is one of the first studies conducted in Portugal linking several databases. During the COVID-19 pandemic, several data linkage studies were conducted [32,33].

To our knowledge, this is the first population-based study that gathers data on mild, moderate, and severe morbidity and mortality among children under 5 years of age and addresses the nonspecific effects of BCG.

In Portugal, this is the first study to assess the impact of the BCG vaccination policy change on the health of children.

### Conclusions

The results will contribute to the accumulating body of knowledge and might have relevance to guide global BCG vaccination policy. The use of data linkage can also contribute to a swifter mechanism to use available health data to conduct population-based studies and inform policy decision-making.

## Appendix

Multimedia Appendix 1 Data on variables (variables requested per database, variables provided per database, number of records in original data sets, actions taken, and final number of records to be linked).

Multimedia Appendix 2 Peer-review report from the Fundação para a Ciência e Tecnologia.

## Abbreviations

BCG: Bacillus Calmette-Guérin vaccine
BDMH: Base de Dados de Morbilidade Hospitalar
DRG: diagnosis-related group
IHMT: Instituto de Higiene e Medicina Tropical
IMR: infant mortality rate
ITQB: Instituto de Tecnologia Química e Biológica António Xavier
MDC: major diagnostic category
NHS: National Health Service
PHC: primary health care
SINAVE: Sistema Nacional de Vigilancia Epidemiologica
TB: tuberculosis
UI: unique identifier

## Data Availability

The data sets analyzed during this study are not publicly available owing to Portuguese Law but are available from the corresponding author on reasonable request.
