# Supplementary material for: Nonspecific Effects of the Bacillus Calmette-Guérin Vaccine in Portuguese Children Under 5 Years of Age: Protocol for a Population-Based Historical Birth Cohort Study
Source: JMIR Res Protoc. 2024 Mar 14;13:e55332. doi: 10.2196/55332 (PMC10979328; doi:10.2196/55332)
Supplement: Multimedia Appendix 1 [file resprot_v13i1e55332_app1.docx]

**Multimedia Appendix 1.** Data on variables (variables requested per database, variables provided per database, number of records in original data sets, actions taken, and final number of records to be linked).

| **Database and variables requested** | **Variables provided** | | **Records**  **provided** | **Actions** | **Records for linkage** |
| --- | --- | --- | --- | --- | --- |
| **RNU - Identification** | | | | | |
| UI | 25/10/2023 | ✓ Pseudonymized User Identifier, which allows linkage with other datasets | 1,262,717 | Check for duplicates.  Auto recode of PHC center | 1,262,717 |
| PHC center |  | ✓ |  |  |  |
| Date of birth |  | Replaced by days of life |  |  |  |
| Gender |  | ✓ |  |  |  |
| Parish |  | 🗶 - data protection |  |  |  |
| Municipality |  | 🗶 - data protection |  |  |  |
| District |  | 🗶 - data protection |  |  |  |
| **Death certificates (SICO) - Mortality** | | | | | |
| UI | 8/11/2023 | ✓ Pseudonymized User Identifier, which allows linkage with other datasets | 1068 | Check for duplicates.  Auto recode of variables place and cause of death | 356 |
| Date of birth |  | 🗶 - data protection |  |  |  |
| Gender |  | ✓ |  |  |  |
| Parish |  | 🗶 - data protection |  |  |  |
| Municipality |  | 🗶 - data protection |  |  |  |
| District |  | 🗶 - data protection |  |  |  |
| Date of death |  | Days from birth until death |  |  |  |
| Place of death |  | ✓ |  |  |  |
| Cause of death |  | ✓ |  |  |  |
| **Vaccines – Registry of all vaccines administered** | | | | | |
| UI | 25/10/2023 | ✓ Pseudonymized User Identifier, which allows linkage with other datasets | 12,011,670 | Identification of duplicates  Restructuring of dataset (link several episodes to one case)  Auto recode of type of vaccine | 975,892 |
| Date of birth |  | 🗶  Replaced by born before 2016 (Y/N) |  |  |  |
| Gender |  | ✓ |  |  |  |
| Parish |  | 🗶 - data protection |  |  |  |
| Municipality |  | 🗶 - data protection |  |  |  |
| District |  | 🗶 - data protection |  |  |  |
| BCG strain |  | 🗶 - not available |  |  |  |
| Type of vaccine |  | ✓ BCG, DTPa, DTPaHib, DTPaHibVIP, DTPaHibVIPVHB, DTPaVIP, DTPaVIPVHB, DTPw, DTPwHib, DTPwHibVIP, DTPwHibVIPVHB, DTPwVHB, Hib, MenC, Pn13, Rotavirus, VASPR, VHB, VIP |  |  |  |
| Date of administration of vaccine |  | Days from birth to administration per dose and type of vaccine |  |  |  |
| Number of doses |  | ✓ per type of vaccine |  |  |  |
| **Sclinico – Mild morbidity – NHS PHC visits not concerning normal surveillance of child health** | | | | | |
| UI | 25/10/2023 | ✓ Pseudonymized User Identifier, which allows linkage with other datasets | 18,952,166 | Identification of duplicates  Restructuring of dataset (link several episodes to one case) | 911,143 |
| Gender |  | ✓ |  |  |  |
| Parish |  | 🗶 - data protection |  |  |  |
| Municipality |  | 🗶 - data protection |  |  |  |
| District |  | 🗶 - data protection |  |  |  |
| Date of the event(s) |  | ✓ per event |  |  |  |
| Cause(s) |  | ✓ Codes ICPC-2 (A01-A99, B01-B99, D01-D99, F01-F99, H01-H99, K01-K99, L01-L99, N01-N99, R01-R99, S01-S99, T01-T99, U01-U99, X01-X99, Y01-Y99) |  |  |  |
| **BDMH – Moderate (emergency department visits without hospital admissions) and severe (hospital admissions) morbidity** | | | | | |
| UI | 17/11/2023 | ✓ Pseudonymized User Identifier, which allows linkage with other datasets | Three separate data sets | Identification of duplicates  Restructuring of dataset (link several episodes to one case) | Three separate data sets |
| Gender |  | ✓ |  |  |  |
| Parish |  | 🗶 - data protection |  |  |  |
| Municipality |  | 🗶 - data protection |  |  |  |
| District |  | 🗶 - data protection |  |  |  |
| Emergency department visit(s) | 17/11/2023 | ✓ | 1,136,058 |  |  |
| Date of episode(s) |  | ✓ |  |  |  |
| Duration of emergency department episode(s) |  | ✓ in days |  |  |  |
| Cause for emergency department episode(s) |  | ✓ ICD-9 or ICD10 codes |  |  |  |
| Outcome of emergency department episode(s) |  | ✓ |  |  |  |
| Hospitalization (s) | 17/11/2023 | ✓ | 1,622,721 |  |  |
| Duration of hospitalization(s) |  | ✓ |  |  |  |
| Cause of hospitalization (s) |  | ✓ ICD-9 or ICD10 codes |  |  |  |
| Outcome of hospitalization (s) |  | ✓ |  |  |  |
| Weight at birth | 17/11/2023 | ✓ | 491,551 |  |  |
| Hospital birth |  | ✓ in Y/N |  |  |  |
| Multiple pregnancy |  | ✓ in Y/N |  |  |  |
| Type of birth |  | ✓ in vaginal or c-section |  |  |  |
| Complication of pregnancy |  | ✓ in Y/N |  |  |  |
| Complication of delivery |  | ✓ in Y/N |  |  |  |
| **SINAVE** | | | | | |
| UI | Contacted to validate variables and data extraction sample (27/11/2023) | IU  Number of cases  Type of case | Data not yet provided | - | - |
| Gender |  | ✓ |  |  |  |
| Parish |  | 🗶 data protection |  |  |  |
| Municipality |  | ✓ |  |  |  |
| District |  | ✓ |  |  |  |
| Date of diagnosis |  | 🗶 Days from birth to notification |  |  |  |
| Date of notification |  | 🗶 Days from birth to diagnosis |  |  |  |
| **SVIG-TB** | | | | | |
| Date beginning of symptoms | Access to SVIG-TB was not granted. Variables were replaced by SINAVE variable (equal or proxy) | 🗶 Days from birth to beginning of symptoms (from SINAVE) | No access to database. Some variables replaced by SVIG-TB | | |
| Date of first appointment |  | 🗶 Not available in SINAVE |  |  |  |
| Detection |  | 🗶 Not available in SINAVE |  |  |  |
| Risk group |  | 🗶 Risk factors 1 and Risk factors 2 |  |  |  |
| Date first dose BCG |  | 🗶 BCG vaccine (Y/N) (from SINAVE) |  |  |  |
| Last dose BCG |  | 🗶 Days from birth to last dose of BCG (from SINAVE) |  |  |  |
| BCG scar |  | 🗶 |  |  |  |
| Mantoux |  | 🗶 |  |  |  |
| IGRA |  | 🗶 |  |  |  |
| Clinical presentation |  | ✓ Presentation of TB  + Signs, symptoms, and images |  |  |  |
| Main location |  | ✓ Primary location  + Secondary location |  |  |  |
| Additional variables provided from SINAVE | | + HIV/aids (Y/N) |  |  |  |
|  |  | + Chronic Pulmonary Disease (Y/N) |  |  |  |
|  |  | + Imunologic medication (Y/N) |  |  |  |
|  |  | + Cancer (Y/N) |  |  |  |
|  |  | + Community housing (Y/N) |  |  |  |
|  |  | + Under 5 child (Y/N) |  |  |  |
|  |  | + Hospital admission (Y/N) |  |  |  |
|  |  | + HIV test (Y/N) |  |  |  |
|  |  | + days of life until death |  |  |  |
| **Legend:** Y/N – yes/no; BCG – Bacile Calmette Guerin vaccine; DTP (1-5) – Diphtheria, tetanus and pertussis vaccine (dose 1 to 5); DTPa - Diphtheria, tetanus and pertussis acellular vaccine; DTPaHib – Diphtheria, tetanus and pertussis acellular vaccine + Haemophilus influenzae type B vaccine; DTPaHibVIP – Diphtheria, tetanus and pertussis acellular vaccine + Haemophilus influenzae type B vaccine; DTPaVIP Diphtheria, tetanus and pertussis acellular + injectable polio vaccine; DTPaVIPVHB – Diphtheria, tetanus and pertussis acellular vaccine + injectable polio vaccine + hepatitis B vaccine; DTPw – Diphtheria, tetanus and pertussis vaccine whole cell; DTPwHib – Diphtheria, tetanus and pertussis whole cell vaccine + Haemophilus influenzae type B vaccine; DTPwHibVIP – Diphtheria, tetanus and pertussis whole cell vaccine + Haemophilus influenzae type B vaccine + injectable polio vaccine; DTPwHibVIPVHB - Diphtheria, tetanus and pertussis whole cell vaccine + Haemophilus influenzae type B vaccine + injectable polio vaccine + hepatitis B vaccine; DTPwVHB – Diphtheria, tetanus and pertussis whole cell vaccine + hepatitis B vaccine; Hib – Haemophilus influenzae type B vaccine; MenC - Meningococcal C vaccine; Pn13 - Pneumococcal conjugate vaccine, Rotavirus – Rotavirus vaccine, VASPR – measles, epidemic mumps and rubella vaccine; VHB - hepatitis B vaccine, VIP - injectable polio vaccine; IGRA - Interferon-Gamma Release Assays. | | | | | |
