# Supplementary material for: Nonspecific Effects of the Bacillus Calmette-Guérin Vaccine in Portuguese Children Under 5 Years of Age: Protocol for a Population-Based Historical Birth Cohort Study
Source: JMIR Res Protoc. 2024 Mar 14;13:e55332. doi: 10.2196/55332 (PMC10979328; doi:10.2196/55332)
Supplement: Multimedia Appendix 2 [file resprot_v13i1e55332_app2.pdf]

PUBLICAÇÃO RESULTADOS AVALIAÇÃO - Candidaturas a Projectos de I&D (INVESTIGADORES) (EVALUATION RESULTS - R&D Projects (RESEARCHERS)

Evaluation Results

Project: EXPL/SAU-EPI/0067/2021

Title: Efeitos não específicos da vacina BCG em crianças com menos de 5 anos de vida

FCT's decision (*Artigo 11º* do Regulamento)

Recommended for Funding - Total Funding: **€49.504,40**

Evaluation Panel Statement and Rating

Subcriterion A1 - Scientific merit of the project from an international standpoint; This subcriterion is intended to evaluate the scientific merit of the proposal, considering the following dimensions in an integrated way:
• Clear identification of the project objectives and scientific challenges addressed by the proposal and its alignment with any of the 2030 Agenda Goals;
• Potential contribution of the research project to the advancement of knowledge.

- **Rating**: 8.50
- **Comments**: This is a very well written project addressing, timely, a question with clear Public Health relevancy. This project sets out to address the important question of whether BCG immunisation confers reduction in mortality and morbidity in infants, which is possible due to the change in vaccination policy implemented in 2016 in line with the change to international guidelines (ie move from vaccinating all children as close to birth as possible to vaccinating high risk groups only). The project objectives and scientific challenges are outlined clearly and properly aligned with UN''s SDG #3 and priorities in child health, immunization and TB. The context of the work in the field is clearly described, establishing the importance and novelty of this project. The fluctuations in infant mortality rates between 2016-19 also give credence to the concept that BCG confers non-specific effects in addition to protection against severe TB. The first cohort of children are now reaching age 5y, thus providing a natural experiment opportunity to determine the impact of the change in vaccination policy on all cause mortality, as well as assess the efficacy of different BCG strains. Data

linkage techniques will be employed to produce a comprehensive database to interrogate and these data will be made available to the scientific community via ORD.

Subcriterion A2 - Innovative nature of the project from an international standpoint;
The present subcriterion is intended to assess the innovative nature of the proposal, considering the following aspects:
• Potential for breakthrough findings by comparison with the current state-of-the-art of the scientific area;
• Methodological innovation, and replication potential;
• Potential impact of the project's outcomes on the economic, technological and societal dimensions.

- **Rating**: 8.50
- **Comments**: This project has a clear potential for breakthrough findings by comparison with the current state-of-the-art in the field. It is well-designed, and timely so, resourcing to the linkage of relevant data sources. The two weaknesses in the project are that the anticipated population and effect size are not described (and the statistical power to address the questions was not provided although there may not be any issues given the large numbers). There are risks associated with the data linkage approach, though these may have been, to some extent, mitigated by the proposed approach and the international scientific advisory with experience of the approach. This project will generate novel and important data with the potential to inform future public health policy. The sharing of the database via the ORD platform will also allow a legacy impact. There is a communication plan including with the Ministry of Health to ensure that the findings inform future vaccination policy.

Subcriterion B1 - Scientific merit of the Principal Investigator;
This subcriterion is intended to evaluate scientific merit of the PI, through the following dimensions:
• Merit of the scientific and professional career of the Principal Investigator valuing the different components: participation in research projects; scientific publications; leadership/organization/participation in networks and conferences; participation in activities of scientific training and management; outreach activities;
• PI´s qualifications regarding the project's challenges, both at the scientific and management level, as well as the ability to engage young researchers in training;
• Relevant outcomes of previous projects and their contribution to the advancement of knowledge and to knowledge-based applications, assessed through the qualitative appraisal of publications or other professional and scientific works and actions considered as the most representative of the of the PI's career.

- **Rating**: 8.50
- **Comments**: The PI is clearly very well equipped and has the experience and skills, both at the scientific and managerial level, to lead this project.

Subcriterion B2 - Scientific merit of the research team.
The present subcriterion is intended to assess the scientific merit of the research team, considering the following aspects:
• Scientific productivity of the team (references to publications and citations in published works, other relevant indicators);
• Ability to engage young researchers in training;
• Degree of internationalisation of the team (when appropriate);
• Abilities and skills to adequately execute the proposed project in its specific area, considering the team's configuration, the availability and commitment of its members (and other entities, when applicable);
• Level of commitment of any companies participating in the project (if applicable).

- **Rating**: 8.50
- **Comments**: The team members blend complementary expertise needed for the successful execution of this project. The investigators have strong records both in terms of publications and conducting similar projects, as well as in supervision of young researchers in training. This is important given the intended involvement of a PhD student in the project (budgeted for). The team will also collaborate with international experts in the field. The involvement of an international Scientific Advisory Board (SAB) will support the delivery of the project by providing data linkage expertise, which is a critical aspect of the project.

Criterion C - Quality and feasibility of the workplan, the expected indicators and the budget reasonability.
This criterion is intended to evaluate the quality and feasibility of the workplan and the expected indicators, as well as the budget adequacy, considering the following aspects:
• Quality (clarity, consistency and adequacy) of the project, taking into consideration the theoretical framework, the research methodology and the work plan;
• Clear identification of the planned activities, their structure and adequacy to the established methods and objectives;
• Adequacy of the human resources and methodologies to perform the proposed objectives and tasks and meet the proposed deadlines;
• If applicable, analysis of the risks associated to the different stages of the project, with special focus on the identification of the critical points and the corresponding contingency plan;
• Valuation of the potential of the predicted outputs (besides other components of the proposal, more detailed information can be found in the application form section 6 "Expected output indicators" and "Knowledge dissemination");
• Adequacy of the physical and financial resources involved in the project, with regard to the host's conditions (technical/scientific, organizational management and, when appropriate, co-funding capacity by companies) provided by the beneficiary entities, in particular institutional resources of the participating entities, namely of the Principal Contractor;
• Adequacy and consistency of the proposed budget to accomplish the objectives and activities proposed.

- **Rating**: 8.00
- **Comments**: The work plan is clearly presented and there is a strong project management. Tasks unfold logically and are appropriate to meet the proposed objectives. Expected outputs seem realistic and represent excellent value for money. The proposed budget is deemed adequate to accomplish the objectives and listed activities. There is a clear risk that may threat the feasibility of the project though, specifically its tide timeline, considering also the shorter duration of EXPL projects: the dependence on the access and linkage of the many data sources, considering also the many administrative, procedural, and scientific complexities this entails and that need to be navigated through. No contingency plan was presented though. It would have been good to provide some assurances on the feasibility (e.g. based on prior experiences in accessing and harmonizing timely those data sources). The communication plan will involve dissemination to scientific peers, policy makers and the public although the latter aspect is not described in detail. This public dissemination is important as may help to combat vaccine hesitancy as highlighted by the research team.
-

Overall Rating: 8.35

Panel Recommended Funding [1]: € 49.504,40

[1] According to budget availability, the total funding recommended by FCT might be different from the one recommended by the panel.
